# Vaccination and Antibody Testing in Cats

**DOI:** 10.3390/v14081602

**Published:** 2022-07-22

**Authors:** Herman Egberink, Tadeusz Frymus, Katrin Hartmann, Karin Möstl, Diane D. Addie, Sándor Belák, Corine Boucraut-Baralon, Regina Hofmann-Lehmann, Albert Lloret, Fulvio Marsilio, Maria Grazia Pennisi, Séverine Tasker, Etienne Thiry, Uwe Truyen, Margaret J. Hosie

**Affiliations:** 1 Department of Biomolecular Health Sciences, Faculty of Veterinary Medicine, University of Utrecht, 3584 CL Utrecht, The Netherlands; 2 Department of Small Animal Diseases with Clinic, Institute of Veterinary Medicine, Warsaw University of Life Sciences-SGGW, 02-787 Warsaw, Poland; tadeusz_frymus@sggw.edu.pl; 3 Clinic of Small Animal Medicine, Centre for Clinical Veterinary Medicine, LMU Munich, 80539 Munich, Germany; Hartmann@lmu.de; 4 Institute of Virology, Department for Pathobiology, University of Veterinary Medicine, 1210 Vienna, Austria; karinmoestl@gmail.com; 5 Maison Zabal, 64470 Etchebar, France; draddie@catvirus.com; 6 Department of Biomedical Sciences and Veterinary Public Health (BVF), Swedish University of Agricultural Sciences (SLU), P.O. Box 7036, 750 07 Uppsala, Sweden; sandor.belak@slu.se; 7 Scanelis Laboratory, 31770 Colomiers, France; corine.boucraut@scanelis.com; 8 Clinical Laboratory, Department of Clinical Diagnostics and Services, Vetsuisse Faculty, University of Zurich, 8057 Zurich, Switzerland; rhofmann@vetclinics.uzh.ch; 9 Fundació Hospital Clínic Veterinari, Universitat Autònoma de Barcelona, Bellaterra, 08193 Barcelona, Spain; albert.lloret@uab.es; 10 Faculty of Veterinary Medicine, Università degli Studi di Teramo, 64100 Teramo, Italy; fmarsilio@unite.it; 11 Dipartimento di Scienze Veterinarie, Università di Messina, 98168 Messina, Italy; mariagrazia.pennisi@unime.it; 12 Bristol Veterinary School, University of Bristol, Bristol BS40 5DU, UK; s.tasker@bristol.ac.uk; 13 Linnaeus Veterinary Limited, Shirley, Solihull B90 4BN, UK; 14 Veterinary Virology and Animal Viral Diseases, Department of Infectious and Parasitic Diseases, FARAH Research Centre, Faculty of Veterinary Medicine, Liège University, B-4000 Liège, Belgium; Etienne.Thiry@uliege.be; 15 Institute of Animal Hygiene and Veterinary Public Health, University of Leipzig, 04103 Leipzig, Germany; truyen@vetmed.uni-leipzig.de; 16 MRC—University of Glasgow Centre for Virus Research, Glasgow G61 1QH, UK; Margaret.Hosie@glasgow.ac.uk

**Keywords:** feline, titre testing, vaccine, immunization, maternal antibodies, duration of immunity, DOI

## Abstract

Vaccines protect cats from serious diseases by inducing antibodies and cellular immune responses. Primary vaccinations and boosters are given according to vaccination guidelines provided by industry and veterinary organizations, based on minimal duration of immunity (DOI). For certain diseases, particularly feline panleukopenia, antibody titres correlate with protection. For feline calicivirus and feline herpesvirus, a similar correlation is absent, or less clear. In this review, the European Advisory Board on Cat Diseases (ABCD) presents current knowledge and expert opinion on the use of antibody testing in different situations. Antibody testing can be performed either in diagnostic laboratories, or in veterinary practice using point of care (POC) tests, and can be applied for several purposes, such as to provide evidence that a successful immune response was induced following vaccination. In adult cats, antibody test results can inform the appropriate re-vaccination interval. In shelters, antibody testing can support the control of FPV outbreaks by identifying potentially unprotected cats. Antibody testing has also been proposed to support decisions on optimal vaccination schedules for the individual kitten. However, such testing is still expensive and it is considered impractical to monitor the decline of maternally derived antibodies.

## 1. Introduction

Vaccination guidelines, as published by the Advisory Board on Cat Diseases (ABCD), aim to support the practitioner in making informed decisions regarding vaccination schedules for an individual animal and/or a group of animals. After primary vaccination, re-vaccinations with the core vaccines for feline calicivirus (FCV) and feline herpesvirus (FHV) are recommended every 1–3 years, depending on the risk of infection and every 3 years with the core vaccine for feline panleukopenia virus (FPV) [1]. Vaccination intervals are based on the minimal duration of immunity (DOI) as determined in experimental vaccination-challenge studies performed by the vaccine industry. However, individual differences exist as to which age kittens can be vaccinated successfully (because maternally derived antibodies to different viruses vary in duration) and how long vaccine-induced DOI lasts. In addition, vaccine-induced immunity in adult cats can be much longer than those DOI determined by industry challenge experiments. Moreover, cats might have undergone subclinical infection and might be protected life-long even without having received any vaccination. Additionally, individual immune reactions and subsequent DOI might vary and depend on many different factors, such as age, nutritional status, concurrent subclinical infections, and breed [2]. To achieve an optimal vaccination schedule for the individual animal and to avoid unnecessary vaccinations, antibody testing can be helpful. However, it is important to differentiate between actively or passively acquired antibodies during the interpretation of antibody testing, including titre testing. While titres of passively acquired antibodies, which generally only persist for weeks, allow a quantitative interpretation about the level of protection (“protective titre”), this is not the case for actively acquired antibodies. Following infection or vaccination antibodies and cellular immune responses are induced through the activation of T and B-cells and the formation of memory cells. The presence of antibodies indicates that an immune response has been induced, irrespective of the antibody titre.

Live vaccines induce a humoral (antibodies) immune response as well as a cell-mediated immune response (CMI). The CMI plays an important role in the control of intracellular pathogens, such as viruses. However, vaccination-challenge experiments have provided excellent data to show that there is also a good correlation between the vaccine-induced antibody titre and protection against certain diseases [3]. Antibody titre as a measure of immunity has been shown to be useful for the core vaccines against canine distemper virus (CDV), canine adenovirus (CAV)-1, canine parvovirus (CPV)-2, and rabies [4,5] in dogs and against FPV and also rabies in cats. For rabies virus vaccination, however, national and regional legislation will determine recommendations for primary vaccinations and revaccinations, and thus, antibody testing is not performed routinely to determine the need for vaccination. For some other vaccine-preventable diseases such as those caused by FCV and FHV, a correlation between antibody titres and protection does not exist, and the role of antibodies is less clear. Protection against FCV has been shown to correlate to humoral virus-neutralising antibodies (VNAs) and CMI [6]. The important role of CMI in FCV is supported by cats being protected against infection despite an absence of detectable VNAs [7,8,9]. The level of mucosal IgA is a stronger correlate of protection than blood antibody levels, but levels of mucosal antibodies cannot be measured easily [10]. Also, because of FCV strain variation in the field, the value of antibody testing in predicting protection is limited [11,12]. Neutralising antibody titres detected against laboratory strains of FCV might not correlate with neutralisation (protection) against field strains due to the absence of, or insufficient, cross-neutralisation.

For FHV infection, as in other alphaherpesvirus infections, CMI is more important than humoral immunity for protection [13]; however, cellular immune responses can only be measured by sophisticated laboratory methods [13]. It has been shown that serum antibody testing is not useful to predict protection against FHV infection [14]. The absence of serum antibody in vaccinated cats does not mean that cats will develop disease. Also, being a pathogen of the respiratory tract, mucosal cellular and humoral responses are important in protecting cats against FHV infection [13]. 

For FeLV infection, high levels of VNAs can be detected in most cats that had overcome viraemia after field infection (but usually not after vaccination), indicating a role in protection, although cytotoxic T-lymphocytes also are very important [15]. Results of a recent study on the humoral immune response in cats with regressive or progressive FeLV infection also support a role for VNAs in protection. VNAs were only detected in the cats with regressive infections, and these cats also showed higher FeLV-antibody responses against the surface protein (gp70) compared to cats with progressive infections [16].

Assays for VNAs are helpful as VNA positive cats are immune to FeLV and do not require vaccination. However, tests to measure VNAs are not widely available. Recently, a new FeLV antibody test became available in some European countries. It is a POC test that measures antibodies to FeLV p15E (envelope transmembrane protein). This new test is based on the results of a study that assessed the diagnostic utility of the detection of antibodies against different FeLV antigens. The recombinant preparation of FeLV p15E was identified as a potentially useful antigen for the detection of antibodies that could correlate with protection [17]. The value of this new POC test to predict protection against FeLV infection, however, has still to be evaluated, and it remains unknown how well the presence of anti-p15E antibodies correlates with protection from FeLV infection in the field.

Since good correlation between antibody titer and protection against disease has been observed for FPV in particular, the focus of this review will be on testing for antibodies against FPV in the context of vaccination. Measurement of the presence of, and/or the levels of, antibodies in a serum sample can be performed by either sending a sample to an external diagnostic laboratory, or by the use of a point-of-care (POC) test that can be used in the veterinary practice (i.e., ‘patient-side’ or ‘in-clinic’ testing).

## 2. Antibodies and Protection against FPV

Results from experimental and field studies in FPV vaccination indicate that antibodies persist for far longer than 3 years; in some animals, lifelong antibody persistence was demonstrated [18,19]. Because of the long DOI raised by the FPV vaccines, cats with sufficient antibodies will not respond to the 3-yearly booster vaccination and, therefore, these animals will be vaccinated unnecessarily. Only cats with no or very low antibody titres will benefit from vaccination. In this regard, the 3-yearly booster interval, as recommended in current guidelines for FPV, is merely intended to sustain herd immunity by ensuring that all unprotected cats are vaccinated. For an individual cat, the antibody level can be determined using either gold standard tests or POC test kits to decide whether a (re)-vaccination is needed. Gold standard tests provide precise antibody titres, whereas POC test kits provide only qualitative (present or absent) or semi-quantitative results (high, medium, low or no antibodies).

### 2.1. Gold Standard Tests

The antibodies that protect against infection are directed against the viral surface proteins and thus prevent the infection of cells. These antibodies are particularly important for the prevention of systemic infections like FPV, their levels can be determined in diagnostic laboratories with a virus neutralisation test (VN) or a haemagglutination inhibition (HI) assay. These tests are considered gold standard methods of determining the titre of protective antibodies in serum.

The antibody titre is determined by making serial dilutions of the serum sample, which are then added to a standard amount of virus. After specific incubation times, the virus-antibody mixture is inoculated onto cell cultures or added to red blood cells. The titre is defined as the reciprocal value of the highest dilution that prevents the infection of cells (VN) or the agglutination of red blood cells (HI assay).

### 2.2. Point-of-Care (POC) Test Kits

Different POC tests are available for use in veterinary clinics. The tests are ELISA- or immuno-migration-based, and results of some of the tests have been validated against the gold standard assays [20]. In one POC test, based on a solid phase dot ELISA, antibodies against FPV, as well as FCV and FHV, are detected, and a maximum of 12 feline samples can be investigated at once. The result can be read by comparing the colour tone of the test spots with the control spot, which gives a semi-quantitative result. Data on the sensitivity and specificity of this test were provided by the commercial company that produced the test and were published also in two independent studies [20,21]. The test kit showed 99% specificity and 49% sensitivity in a study in shelter cats [21]. Mende et al. [20] reported the results of a study after the test had been modified to improve sensitivity. In this study, the test showed 89% specificity and 79% sensitivity when compared with a HI titre at a cut-off titre of 20; this cut-off titre was chosen, as a titre of ≥20 in adult cats that have been vaccinated or have overcome active infection is considered protective [22]. In another study, cats were considered to be protected against FPV if they had a HI titre of ≥40 [19]. At a titre cut-off point of 40, the specificity of the assay was determined as 86% and sensitivity 83%. To identify cats that have insufficient antibody levels and therefore require vaccination, specificity is the more important parameter since it determines the percentage of negative tests that are correctly identified. It is important to minimise the number of false positive results that can be expected because cats with a false positive test result will not receive a booster vaccination and will potentially remain unprotected. The specificity is considered acceptable, assuming a titre of ≥20 is protective. If the test is used in cats belonging to a population with an expected high prevalence of antibody-positive animals, the positive predictive value is high, and the test can be considered suitable for use in veterinary practice, for example in adult cats with known vaccination or infection history [20].

New POC tests that have recently become available that detect antibodies against only FPV (and not FCV or FHV) are based on an immunochromatographic principle and generally deliver qualitative results (i.e., giving a result of protected or not protected rather than a specific antibody titre) in a shorter period of time. These tests have not been evaluated in independent studies so far.

## 3. Applications of POC Antibody Testing against FPV

### 3.1. To Measure the FPV Antibody Response in Kittens Following Vaccination

After the initial series of vaccinations in the first few months of life of a kitten, vaccine-induced protection can be determined by POC tests. As the last vaccination is usually given around the age of 12–16 weeks, a positive test result in an antibody test obtained at the age of 20 weeks indicates that the animal has made an active immune response. At this age, maternally derived antibodies (MDA) are expected to have waned to very low or undetectable titres in the majority of animals [23]. If the last vaccine was given at an age of 16 weeks and protection was shown at 20 weeks, the WSAVA vaccination guidelines state that the 12-month booster might not be required and that animals could go straight to a triennial FPV vaccination program [24]. There is not much data regarding the age at which the immune system matures and if the quality of the induced immune response at 16 weeks is as good as in adult animals. Therefore, it seems valid to advise yearly antibody testing in these animals rather than going straight to a triennial vaccination programme, particularly if the last vaccine was given before the age of 16 weeks. A kitten that is negative for FPV antibodies at the age of 20 weeks should be revaccinated and tested again 3–4 weeks later to determine if antibodies have developed. If the animal is still negative, the kitten is most likely a non-responder to the particular FPV antigen and might be susceptible to infection and disease for life [24].

### 3.2. To Test Whether (Re)Vaccination for FPV Is Necessary

Triennial vaccination for FPV is based on the minimal DOI. Since many vaccinated animals will maintain protective antibody titres for longer than a 3-year period, sometimes even lifelong, triennial antibody testing can be performed as an alternative for routine booster vaccination. Re-vaccinations were shown not to be beneficial especially in cats with high titres [2]. For adult cats with an unknown vaccination history, or elapsed vaccination, an antibody test could be offered to owners as an alternative to revaccination for FPV. However, this requires that monovalent FPV vaccines are available to allow differential administration of specific vaccines, which is not the case in all countries.

In animals that have previously experienced a serious adverse vaccine reaction, the need for revaccination should be carefully evaluated. This holds true for core and non-core vaccines. For FPV vaccines, this decision can be made based on the results of a positive FPV POC antibody test. Another situation in which the requirement for vaccination can be determined by antibody measurement is in immunocompromised cats (see ABCD guidelines on Vaccination of immunocompromised cats [11,25]).

### 3.3. To Control FPV Infection and Disease Outbreak in Shelters

If possible, animals could also be tested for FPV antibodies before admission into a shelter to determine if they are protected. If they are not protected, the animals should be vaccinated and kept in strict isolation or preferably sent to foster homes to develop active immunity before entering the shelter. However, it is recognised that in shelters, routine antibody screening might not be appropriate because of the extra costs; therefore, often the preference is to elect for vaccination as soon as possible after entry.

In the face of an outbreak of FPV disease, susceptible cats without FPV antibodies can be identified using the POC antibody test and can then be immediately vaccinated or receive hyperimmune serum (passive immunization). The advantage of such an approach is that protected antibody-positive animals can then be separated from the cats without antibodies. Antibody-positive animals do not need to be vaccinated. The antibody-negative animals, following vaccination, should be isolated at least until the incubation period of the disease has passed (on average 2–7 days). These animals should be retested before adopting out. Passive immunization of these unprotected cats also might be a short-term option. In countries where they are available, commercial immunoglobulin preparations containing antibodies against FPV can be used. Also, homologous immune serum from blood of cats with high antibody levels can be prepared and administered. Blood donors must be screened for insidious agents (e.g., FIV, FeLV, *Bartonella* infection) and attention must be paid to sterility, storage, and administration. Also, the blood type of a donor and recipient should match [26].

### 3.4. To Determine the Optimal Age of Primary Vaccination for FPV in Kittens

#### 3.4.1. Acquisition of FPV MDAs

During the first period of their lives, kittens are protected by MDAs. Cats have an endotheliochorial placenta that is a barrier to immunoglobulins, preventing their passage from the maternal serum into the foetal circulation. It is generally believed that only up to 5–10% of the MDAs are transferred during pregnancy from an immune queen to the foetuses [27,28,29,30]. Claus et al. (2006) found that none of 182 neonatal kittens had detectable IgG or IgA serum levels at parturition [30]. This confirms that in cats the vast majority, if not all, of MDA is transferred to the offspring via colostrum, which contains trypsin inhibitors that protect immunoglobulins from degradation in the gastrointestinal tract of the newborn. The dominating immunoglobulin class in colostrum is IgG, which is concentrated 2–5 fold in the mammary glands from the serum of the queen [30,31].

Mammalian neonates absorb intact colostral immunoglobulins from the intestinal lumen into circulation. However, this ability decreases rapidly and generally ceases by 24 h after birth. A neonatal kitten is able to absorb immunoglobulins most efficiently within the first 16 h of life [32]. The higher the levels of antibodies in the colostrum, the more likely the kitten will receive adequate levels of antibodies from its mother. The transfer of MDA is so efficient that some kittens achieve higher levels of circulating antibodies than their mothers [30,33].

Several factors can impair the transfer of MDA to neonates, resulting in hypogammaglobulinemia in the kittens, such as delayed onset of nursing and poor colostrum intake within the first 16–24 h after birth. Therefore, orphaned neonates or those rejected before nursing (as well as those removed before nursing to avoid neonatal isoerythrolysis) are at risk, as well as kittens from very large litters, small or weak neonates, and offspring from queens that fail to produce colostrum with adequate immunoglobulin content. Failure of MDA transfer leads to a very high risk of sepsis or other severe infection and death within the first 2 weeks of life. Hypogammaglobulinaemia in a kitten older than 16–24 h can be corrected by s.c. or i.p. administration of 3–5 mL of serum from a healthy, properly vaccinated cat, preferably living in the same environment as the affected kittens [34]. Attempts to replace feline serum by equine IgG have been unsuccessful [35].

Immunoglobulins are present not only in colostrum, but also in feline milk. These are, however, not derived from the mother’s serum, but produced in the milk gland. Such lactogenic immunity provides local protection on the mucosal surfaces of the offspring, especially in the gastrointestinal tract, and seems to play a protective role until weaning. These secretory immunoglobulins are also present in the colostrum and are absorbed into the circulation of the newborn before being released back onto mucosal surfaces.

#### 3.4.2. Duration of Protection by MDAs

Passively acquired, circulating immunoglobulins provide immediate systemic protection that is essential during the early life of the kittens. As in all mammals, passive immunity in cats is very effective but only short lasting. Circulating MDAs are catabolized by the neonate, thus their concentration gradually decreases with age. In kittens, the serum IgG levels decline continuously from a peak on day 2, reaching a nadir between the ages of 4–5 and 16 or more weeks of age. The period of this elimination depends strongly on the initial MDA level in the circulation of the neonate after colostrum uptake, environmental factors, and the respective antigens. As a result, there are great differences between kittens (and even between littermates) in the period of protection afforded by circulating MDA.

The period of MDA elimination for FPV has been assessed as 8–14 weeks [27], although more recent studies suggest that MDA can persist until the age of 16 or even 20 weeks [36]. For comparison, this period encompasses the first 4–6 weeks of life for feline coronavirus (FCoV) [37], 6–8 weeks for FeLV [38] and FHV [39,40], 10–14 weeks for FCV [41], and 12 weeks for feline immunodeficiency virus (FIV) [42].

#### 3.4.3. Active Immunization in Early Life

As soon as maternal antibodies decrease to unprotective levels, kittens need to be vaccinated to induce an active immune response. It is generally believed that, similar to most mammals, kittens are able to generate an immune response to antigens from birth, but, compared to adult animals, this response is impaired unless the immune system is mature [43]. It has been demonstrated that IL-2 production after Concanavalin A stimulation is much lower in kittens of less than 10 weeks of age compared to adult animals [44]. Another example of immune system maturation is the development of age-related resistance to feline leukemia virus (FeLV) infection in kittens. An experimental infection resulted in persistent viraemia and FeLV-related disease in 100% of newborns, in 85% of kittens inoculated at 2 weeks to 2 months of age, but in only 15% of cats infected at 4 months or 1 year of age [45].

MDAs protect animals from infection but also interfere with the development of post-vaccinal immunity [23]. Very young kittens can generate an effective antibody response to vaccination only when the levels of MDA against the respective antigen have decreased below the inhibitory threshold. The level of MDAs will differ between litters and individual animals within litters, depending on the antibody levels in the colostrum of the queens and the amount of colostrum ingested. Therefore, it is common practice to perform the first core vaccination at 8–9 weeks of age (or earlier in kittens at special risk or in rescue shelters), and to give additional doses at 2- to 4-week intervals until the age of 12–16 weeks or older [46], with the expectation that one of these vaccinations will fall after the blocking effect of the MDA and before exposure to virulent agents. With this strategy, some doses might be given that are of no benefit if the kitten still has interfering levels of MDAs or if the kitten has already responded to an earlier vaccine (or infection) and thus is already immune.

To avoid this problem, in an ideal world, the optimal age for the first core vaccination would be determined by establishing the antibody titre of each kitten to determine when interfering MDAs have waned. However, the use of antibody testing in this situation has not yet been critically evaluated. If using a POC test that provides no titres but only semi-quantitative results, kittens would likely need to be re-tested every 2–3 weeks since the optimal time point for vaccination might not be determined by a single blood sample at an age of 6–8 weeks. It is recognized that repeated blood sampling of young kittens every 2–3 weeks is difficult and potentially stressful for the kittens, as well as costly, precluding routine adoption of this procedure in practice.

Data defining the levels of MDAs at which vaccination will lead to active immunization are lacking. Also, differences in the performance of available vaccines in the presence of MDAs can be expected. Where antibody testing is being used to decide upon the optimal age of first vaccination, a titre, as produced by diagnostic laboratories, is preferable to the semi-quantitative result of a POC test given the increased precision of the result. However, antibody testing is still expensive and often impractical in this situation.

An estimate of the optimal age for first vaccination of kittens can also be calculated based on the antibody titre of the queen, and the average half-life of MDAs (9.5 days for FPV MDA), bearing in mind that individual kittens in a litter will suckle different amounts of colostrum [47]. 

## 4. Conclusions

Determining the optimal age of immunization of kittens by antibody testing with POC tests can be problematic in the face of decreasing maternal antibody titres for reasons described above. In contrast, antibody (titre) testing, especially against FPV, can be a useful and reliable tool to determine whether a cat has developed antibodies after vaccination, and to determine whether the individual animal needs revaccination at the time proposed in the general vaccination guidelines. In vaccinated adult cats, titre testing can be conducted as part of the annual health check appointment, which could also include a complete blood count, serum biochemistry, and urinalysis, at least in mature animals. Since data on the role of ageing of the immune system on the persistence of levels of antibodies are lacking, yearly testing in older cats (>15 years) is strongly advised. This guideline will continue to be updated regularly on the ABCD homepage (www.abcdcatsvets.org, accessed on 15 March 2022) as new data become available.
